# Body composition and perceived stress levels in transgender individuals after one year of gender affirming hormone therapy

**DOI:** 10.3389/fendo.2024.1496160

**Published:** 2024-11-28

**Authors:** Chiara Ceolin, Alberto Scala, Bianca Scagnet, Anna Citron, Federica Vilona, Marina De Rui, Marina Miscioscia, Valentina Camozzi, Alberto Ferlin, Giuseppe Sergi, Andrea Garolla, the GIIG group

**Affiliations:** ^1^ Geriatrics Division, Department of Medicine (DIMED), University of Padua, Padua, Italy; ^2^ Department of Neurobiology, Care Sciences and Society, Karolinska Institutet and Stockholm University, Aging Research Center, Stockholm, Sweden; ^3^ Regional Reference Center for Gender Incongruence (CRRIG) of the Veneto Region, University Hospital of Padova, Padua, Italy; ^4^ Unit of Andrology and Reproductive Medicine, Department of Medicine (DIMED), University of Padua, Padua, Italy; ^5^ Department of Developmental Psychology and Socialization, University of Padova, Padua, Italy; ^6^ Department of Women’s and Children’s Health, University of Padova, Padua, Italy; ^7^ Endocrinology Unit, Department of Medicine (DIMED), Azienda Ospedale-Università Padova, Padua, Italy

**Keywords:** transgender, stress, body composition, bone mineral density, PHQ, PSS

## Abstract

**Introduction:**

Higher stress levels are linked to increased body fat and decreased bone density, effects that can be exacerbated by lifestyle choices. This is particularly relevant for transgender and gender diverse (TGD) individuals, who often face additional stress from transphobia and social stigma. However, there is limited research on how stress affects body composition and bone health in TGD individuals, particularly in relation to gender-affirming hormone therapy (GAHT). This study examines the impact of perceived stress on these factors in TGD individuals before and after one year of GAHT, compared to a cisgender control group.

**Methods:**

The study assessed 181 individuals, including 74 TGD participants (44 assigned female at birth [AFAB] and 30 assigned male at birth [AMAB]) and 107 controls (56 AFAB and 51 AMAB). Fifty-seven TGD participants completed follow-up one year after starting GAHT. Data collected included clinical history, blood tests, body composition, bone density, and quality of life assessments (Patient Health Questionnaire-9 [PHQ-9] and Perceived Stress Scale [PSS]).

**Results:**

After one year of GAHT, TGD AFAB individuals showed a bone mineral density (BMD) similar to cisgender AMAB individuals, while TGD AMAB individuals’ BMD remained significantly lower than cisgender controls. TGD AFAB individuals experienced increases in muscle strength (+8% from baseline), while TGD AMAB individuals showed a 24% increase in fat mass from baseline and an approximate 8% reduction in lean mass. PSS and PHQ scores, initially higher in TGD individuals, did not change significantly after one year of GAHT. A significant correlation was found between body fat percentage and PHQ and PSS scores at baseline and one year after GAHT, respectively.

**Discussion:**

These findings reveal a complex relationship between GAHT, body composition, and perceived stress in TGD individuals, highlighting the need for further research on stress and health outcomes in this population.

## Graphical Abstract

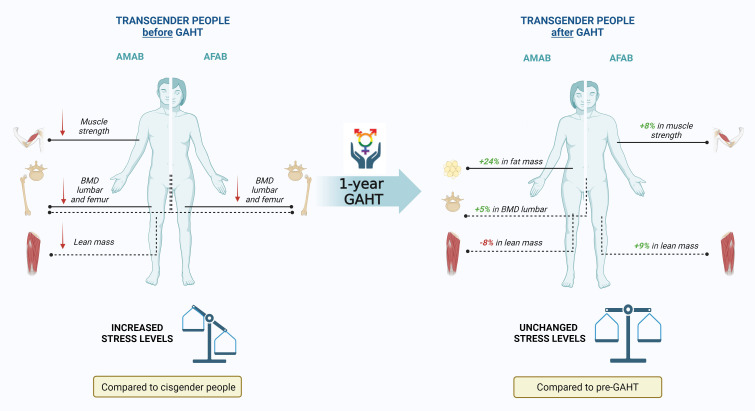

## Introduction

Gender and biological sex are two dimensions of sexual identity that should not be overlapped. Individuals, are assigned male at birth (AMAB) or female at birth (AFAB) based on their external genitalia (1). However, transgender individuals experience a mismatch between their gender identity and the sex assigned at birth, a condition known as “Gender Incongruence” (GI) (1). Transgender and gender-diverse (TGD) individuals may identify as men, women, or along a spectrum of alternative gender identities (e.g. non-binary) (1, 2). The prevalence of TGD individuals seeking medical interventions to affirm their gender is estimated between 0.005% and 0.014% of the population (3). Gender-affirming medical and surgical treatments (GAMST) aims to align bodily characteristics with the individual’s gender identity. For TGD individuals, gender-affirming hormone therapy (GAHT) typically involves administering testosterone to AFAB individuals or a combination of estrogen and anti-androgens to AMAB individuals (2).

Sex hormones play a crucial role in the distribution of body mass, affecting both fat and lean tissue, as well as influencing bone health. Typically, female individuals tend to have a higher proportion of adipose tissue, whereas male individuals exhibit greater muscle mass (4). Consequently, it is expected that the administration of GAHT will affect the body composition of TGD individuals during their gender-affirming pathway. To date, studies reported that TGD AMAB people, even before hormone therapy, have lower muscle mass and strength compared to cisgender male controls (5). During GAHT, their body composition tends to align more closely with cisgender women, with an increase in fat mass and body mass index (BMI) and a decrease in lean mass (6). For TGD AFAB individuals, testosterone therapy is associated with an increase in lean mass. Several studies have also demonstrated associations between testosterone treatment and a decrease in fat mass, particularly subcutaneous fat (6). At the same time, it has been reported that TGD AMAB individuals typically have lower bone mineral density (BMD) and bone mineral content (BMC) before starting GAHT compared to their cisgender counterparts (5, 7). This is often accompanied by altered bone geometry (8). In contrast, no significant changes in bone structure and density have been observed in TGD AFAB individuals (9). The administration of estrogen can occasionally result in an increase in bone mineral density (BMD), though the findings are still inconclusive (10).

It is well-established that several factors can influence body composition and bone strength, including physical activity levels, diet, and stress. For instance, research has consistently highlighted a connection between stress levels and fat deposition, demonstrating that higher stress is associated with increased BMI, larger body circumference measurements, and a higher percentage of body fat (11). Furthermore, chronic stress negatively affects bone density and quality (12). This relationship affects individuals across various age groups, with lifestyle factors playing a moderating role (11–13). Due to historical and current systemic and internalized stigma, TGD people can experience significant and different levels of distress. When seeking healthcare, they often encounter ignorance and barriers to accessing services, problems that are exacerbated by discrimination and prejudice (1). This context of marginalization leads to a specific form of stress known as “minority stress” (14). Originally developed to explain health disparities among sexual minorities, such as lesbian, gay, and bisexual individuals, the minority stress model has been adapted to include the experiences of TGD people (15, 16). A key concept in this context is cisnormativity, which refers to the assumption that a person’s gender identity and gender expression should align with the sex assigned at birth (15). This view, which considers cisgender identities as the norm, contributes to the marginalization of TGD individuals. Gender minority stress has significant effects not only on mental health but also on physical well-being of TGD people (17). For instance, studies have shown that TGD individuals may engage in less healthy behaviors, such as reduced physical activity, higher consumption of tobacco and alcohol, and less balanced diets, all of which can negatively impact bone health and other aspects of physical well-being (18). Despite these concerning findings, to the best of our knowledge, there are no studies that have specifically examined the impact of stress on body composition and bone health in TGD individuals, either prior to or during GAHT.

Therefore, in light of these considerations, the aim of this study is to assess the effect of perceived stress on body composition and bone health in TGD individuals both before the initiation of GAHT and one year after starting hormonal therapy. The results will be compared with those from a control group of cisgender individuals.

## Materials and methods

### Study design

The study “Body COmposition and Bone MEtabolism in Transgender adults” (COMET) is an observational study carried out at the University Hospital of Padua (Italy), involving both TGD and cisgender participants.

### Study population

TGD individuals were evaluated at the Regional Reference Center for Gender Incongruence (CRRIG) at the University Hospital of Padua. Cisgender volunteers were recruited from the hospital and university staff in Padua through a targeted selection process. The inclusion criteria for the study have been previously published (7). Briefly, participants experiencing GI, who had not yet started any GAMST, aged between 20 and 50 years, and with a BMI between 19 and 35 kg/m² at the time of inclusion, were eligible. Exclusion criteria included chronic use of medications that affect bone metabolism (such as glucocorticoids, thyroxine, immunosuppressants, NSAIDs, PPIs, diuretics, vitamin D, and calcium), a history of hyperparathyroidism, and use of hormonal contraceptives.

The study protocol was approved by the Local Ethics Committee (Ethics Committee for Clinical Experimentation of the Province of Padua, number 0025087) and adheres to the guidelines of the Declaration of Helsinki. Each participant provided written informed consent to take part in the research.

### Data collection

Each participant underwent comprehensive data collection, including:

#### Patient characteristics

Detailed physiological, clinical, and pharmacological information was collected for each participant, covering factors such as tobacco use.

##### Anthropometry

Body weight and height were measured with participants wearing light clothing and no shoes. BMI was calculated as body weight in kilograms divided by height in square meters.

##### Laboratory data

Fasting blood samples were collected in the early morning to enable the analysis of various parameters, such as estradiol, total testosterone, luteinizing hormone (LH), follicle-stimulating hormone (FSH), serum calcium, phosphate, parathyroid hormone (PTH), and 25-hydroxy-vitamin D (25-OH-D). All laboratory analyses were conducted following standardized procedures at the Laboratory Medicine Unit of the University Hospital of Padua.

##### Bone measurements

We employed Dual Energy X-ray Absorptiometry (DXA) utilizing fan-beam technology (Hologic QDR 4500 W, Inc.) to measure BMD at the proximal femur (femoral neck and/or total hip) and lumbar spine for each patient. Complete details were previously published (7). A whole-body DXA scan was performed to measure fat-free mass (FFM), fat mass (FM), and appendicular skeletal muscle mass (ASMM). The fat mass index (FMI) and appendicular skeletal muscle mass index (ASMMI) were calculated by dividing FM and ASMM by height in square meters. Furthermore, peripheral Quantitative Computed Tomography (pQCT) was utilized to enhance the assessment of bone composition. This involved scanning the right tibia using the Norland/Stratec XCT-3000 scanner (Stratec Medizintechnik GmbH, Pforzheim, Germany). A standardized protocol was followed for positioning and scanning participants, beginning with a scout view to establish an anatomical reference line. Tibia length, measured from the medial malleolus to the medial condyle, provided a crucial anatomical reference. Scans were performed at four key locations along the length of the tibia: 4%, 14%, 38%, and 66%.

From the pQCT images, various bone geometry parameters were derived:

- Trabecular Volumetric Bone Mineral Density (BMDt): This parameter represents the mean density of the trabecular bone area observed at the 4% site on the tibia.
- Cortical Volumetric Bone Mineral Density (BMDc): It signifies the mean density of cortical bone measured at the 38% site on the tibia.
- Total Bone Cross-Sectional Area (CSA): This denotes the area enclosed within the circumference comprising all cortical bone tissues with a density exceeding 180 mg/cm³, evaluated at the 4% site.
- Cortical Bone Cross-Sectional Area (CSAc): This measurement represents the cross-sectional area of the voxels with a density greater than 710 mg/cm³, assessed at the 38% site on the tibia.

##### Psychological well-being questionnaires

- Patient Health Questionnaire-9 (PHQ-9): it consists of 9 items, each scored from 0 (not at all) to 3 (nearly every day). The total score ranges from 0 to 27, with a threshold value of ≥10 used to identify major depression. Major depression is diagnosed if 5 or more of the 9 depressive symptom criteria are present for at least “more than half the days” in the past two weeks, with at least one symptom being depressed mood or anhedonia. “Other depression” is diagnosed if 2, 3, or 4 depressive symptoms are present for at least “more than half the days” in the past two weeks, with at least one symptom being depressed mood or anhedonia (19).
- Perceived Stress Scale (PSS): this self-administered questionnaire assesses how stressful individuals perceive their lives to be. The PSS measures the degree to which individuals believe their life has been unpredictable, uncontrollable, and overloaded in the past month. The temporal validity of stress assessed by the PSS is short, corresponding to 8 weeks (20).

##### Physical activity assessment

- International Physical Activity Questionnaire (IPAQ): details regarding the physical activity assessment were previously reported (7). The IPAQ assesses physical activity levels over the past 7 days, with results expressed in Metabolic Equivalent of Task (MET), a measure of oxygen consumption at rest (3.5 mL O2/kg body mass per minute). MET values vary based on the intensity of physical activity: vigorous (8 MET), moderate (4 MET), and walking (3.3 MET). For this study, METs were categorized based on whether the activities were performed indoors or outdoors.

### Statistical analyses

Categorical variables are expressed as counts and percentages, and continuous quantitative variables as mean ± standard deviation or median (interquartile range-IQR). The normal distribution of continuous variables was verified using the Shapiro-Wilk test. To compare variables between cisgender and TGD individuals or pre- and post-GATH, the Mann-Whitney and Kruskal-Wallis tests were applied for quantitative variables, and the chi-square test was used for categorical variables. Correlations were assessed using Pearson’s correlation coefficient (r) or Spearman’s rank correlation coefficient (rs) when the variables were not normally distributed. In all analyses, significance was assumed at p ≤ 0.05. The analysis was conducted using IBM SPSS Statistics version 29 (IBM Corp., Armonk, NY) and R version 4.1.1 (2021–08–10) (R Foundation for Statistical Computing, Vienna, Austria).

## Results

A total of 181 individuals were evaluated, comprising 74 TGD participants (44 AFAB and 30 AMAB) and 107 controls (56 AFAB and 51 AMAB). About 57 (33 AFAB and 24 AMAB) TGD participants completed a 1-year follow-up after initiating GAHT. **Table 1** presents the baseline general characteristics of the sample. The groups were similar in age, but TGD AFAB individuals had higher BMI values compared to their cisgender peers. Both TGD groups exhibited a notable, although statistically non-significant, tendency toward active smoking. Additionally, TGD AFAB participants showed a tendency to spend less time in outdoor activities, particularly before the initiation of GAHT, compared to the cisgender population [MET for time spent outdoors: 537.00 (90.00; 1282.50) vs. 1170.00 (630.00; 2252.50), data not shown]. Regarding hormonal profiles, no significant differences were observed, except for slightly lower LH levels in TGD AMAB individuals. One year after GAHT, testosterone levels increased in TGD AFAB individuals, while TGD AMAB individuals experienced a decrease in testosterone and an increase in estrogen levels. As a result, TGD people had hormone profiles in line with the desired gender (data not shown).

**Table 1:** Characteristics of the sample at baseline.

Variable	Cis AFAB (n=56)	TGD AFAB (n=44)	p-value	Cis AMAB (n=51)	TGD AMAB (n=30)	p-value
**Age**	25.7 (3.8)	24.2 (5.9)	0.10	25.8 (4.2)	24.9 (7.3)	0.44
**Weight [Kg]**	60.92 (10.29)	68.87 (15.57)	0.05	74.77 (10.10)	72.91 (12.55)	**<0.001**
**BMI [Kg/m^2^]**	21.79 (2.45)	23.75 (4.66)	**0.04**	23.34 (2.48)	23.28 (4.08)	0.96
**Active smokers**	16 (28.6%)	20 (45.4%)	0.08	17 (33.3%)	10 (33.3%)	0.05
Hormonal profile						
**LH [U/L]**	8.90 (5.67;12.85)	6.29 (4.47;9.19)	0.08	5.95 (4.72;7.17)	4.85 (3.52;5.37)	**0.03**
**FSH [U/L]**	5.20 (4.37;5.77)	5.10 (3.20;6.56)	0.49	2.40 (1.95;4.10)	3.95 (1.75;5.84)	0.29
**Estrogens [pmol/L]**	273.50 (169.00;447.75)	226.30 (144.40;494.99)	0.47	100.50 (73.50;136.00)	106.00 (77.05;120.41)	0.91
**Testosterone [nmol/L]**	1.43 (1.04;1.73)	1.10 (0.84;1.69)	0.31	18.90 (15.70;22.25)	19.00 (14.54;22.62)	0.97

Numbers are expressed as mean (standard deviation), number (percentage) or median (interquartile range), as appropriate.

TGD, Transgender; AFAB, Assigned Female At Birth; AMAB, Assigned Male At Birth; BMI, Body Mass Index; LH, Luteinizing Hormone; FSH, Follicle-Stimulating Hormone. Significant p-values are reported in bold.

PSS and PHQ scores, which differed significantly at baseline between TGD and cisgender individuals, did not exhibit significant changes during the first year of GAHT (**Table 2**).

**Table 2:** PSS and PHQ-9 scores after one year of GAHT, compared to the reference cisgender population.

Variable	TGD AFAB		AMAB Cisgender	TGD AMAB		AFAB Cisgender
	Baseline	1-year GAHT		Baseline	1-year GAHT	
**PHQ-9**	10.15 (6.85)	10.90 (6.16)	5.43 (3.26)^§§§^	7.64 (4.78)	8.79 (5.42)	4.67 (3.69)^§§§^
**PSS**	19.05 (8.27)	18.00 (6.36)	13.51 (5.75)^§^	18.21 (5.72)	18.43 (6.06)	15.68 (6.19)^§^

Numbers are expressed as mean (standard deviation).

TGD, Transgender; AFAB, Assigned Female At Birth; AMAB, Assigned Male At Birth; PSS, Perceived Stress Scale; PHQ, Patient Health Questionnaire. ^§^ refers to comparisons between transgender individuals after 1-year of GAHT and cisgender population, ^§^p<0.05; ^§§§^p<0.001.

**Table 3** presents the bone metabolism-related variables among TGD individuals both at baseline and during GAHT, compared to cisgender peers. After one-year of GAHT, TGD AFAB individuals exhibited BMD values comparable to those of their cisgender AMAB peers. In contrast, TGD AMAB individuals showed a significant increase in lumbar BMD, although BMD values at all sites remained significantly lower than those of cisgender women. No significant differences were observed in bone geometry, except for CSAc, which was increased in TGD AFAB after one year of GAHT. In terms of biochemical findings, both TGD cohorts demonstrated heightened vitamin D levels during the initial year of GAHT, reaching levels akin to their cisgender counterparts.

**Table 3:** Bone metabolism-related variables after one year of GAHT, compared to the reference cisgender population.

Variable	TGD AFAB		AMAB Cisgender	TGD AMAB		AFAB Cisgender
	Baseline	1-year GAHT		Baseline	1-year GAHT	
**BMD total hip [g/cm^2^]**	0.92 (0.13)	0.92 (0.13)	1.04 (0.19)	0.92 (1.13)	0.94 (0.15)	1.04 (0.16)^§§§^
**BMD femur neck [g/cm^2^]**	0.81 (0.12)	0.79 (0.14)	1.01 (0.24)	0.93 (0.15)	0.82 (0.15)	0.97 (0.22)^§§§^
**BMD lumbar spine [g/cm^2^]**	1.02 (0.13)	1.01 (0.12)	1.08 (0.14)	0.97 (0.15)	1.02 (0.15)***	1.09 (0.16)^§^
**CSA [mm^2^]**	1001.20 (167.27)	1074.28 (230.92)	1681.87 (2331.85)	1236.68 (225.83)	1306.69 (404.95)	1121.19 (207.80)
**CSAc [mm^2^]**	281.38 (66.15)	347.15 (82.65)***	356.55 (51.86)	299.42 (55.83)	371.34 (112.11)	360.94 (64.27)
**BMDtrb [mg/cm^3^]**	257.42 (80.77)	295.93 (98.57)	256.56 (55.20)^§^	247.45 (22.80)	279.59 (72.18)	298.80 (87.70)
**BMDcrt [mg/cm^3^]**	1165.67 (24.08)	1162.25 (26.83)	1154.79 (25.44)	1155.65 (22.99)	1176.39 (114.22)	1161.61 (33.39)
**Calcium [mmol/L]**	2.54 (0.13)	2.45 (0.17)	2.40 (0.04)	2.42 (0.08)	2.39 (0.05)	2.41 (0.06)
**Phosphor [mmol/L]**	0.99 (0.43)	0.90 (0.35)	1.01 (0.15)	0.99 (0.18)	1.17 (0.17)	2.40 (0.05)
**PTH [ng/L]**	32.20 (24.65-37.35)	36.20 (27.10-78.40)	28.40 (25.60-37.05)	24.95 (18.87-36.47)	52.00 (11.45-68.30)	24.10 (16.20-35.40)
**Vitamin D [nmol/L]**	45.00 (35.00-67.37)	65.90 (44.25-118.00)*	56.95 (40.00-82.60)	40.75 (27.50-69.25)	66.80 (52.47-115.90)*	53.50 (36.00-75.00)

Numbers are expressed as mean (standard deviation), number (percentage) or median (interquartile range), as appropriate.

TGD, Transgender; AFAB, Assigned Female At Birth; AMAB, Assigned Male At Birth; BMD, Bone Mineral Density; CSA, Cross-Sectional Area; CSAc, Cortical Cross-Sectional Area; BMDtrb, Total Body Bone Mineral Density; BMDcrt, Femoral Neck Bone Mineral Density; PTH, Parathyroid Hormone. Asterisks refer to comparisons between transgender individuals before and after 1-year of GAHT: *p<0.05; **p<0.01; ***p<0.001. ^§^ refer to comparisons between transgender individuals after 1-year of GAHT and cisgender population: ^§^p<0.05; ^§§^p<0.01; ^§§§^p<0.001.Significant p-values are reported in bold.

**Figure 1:**
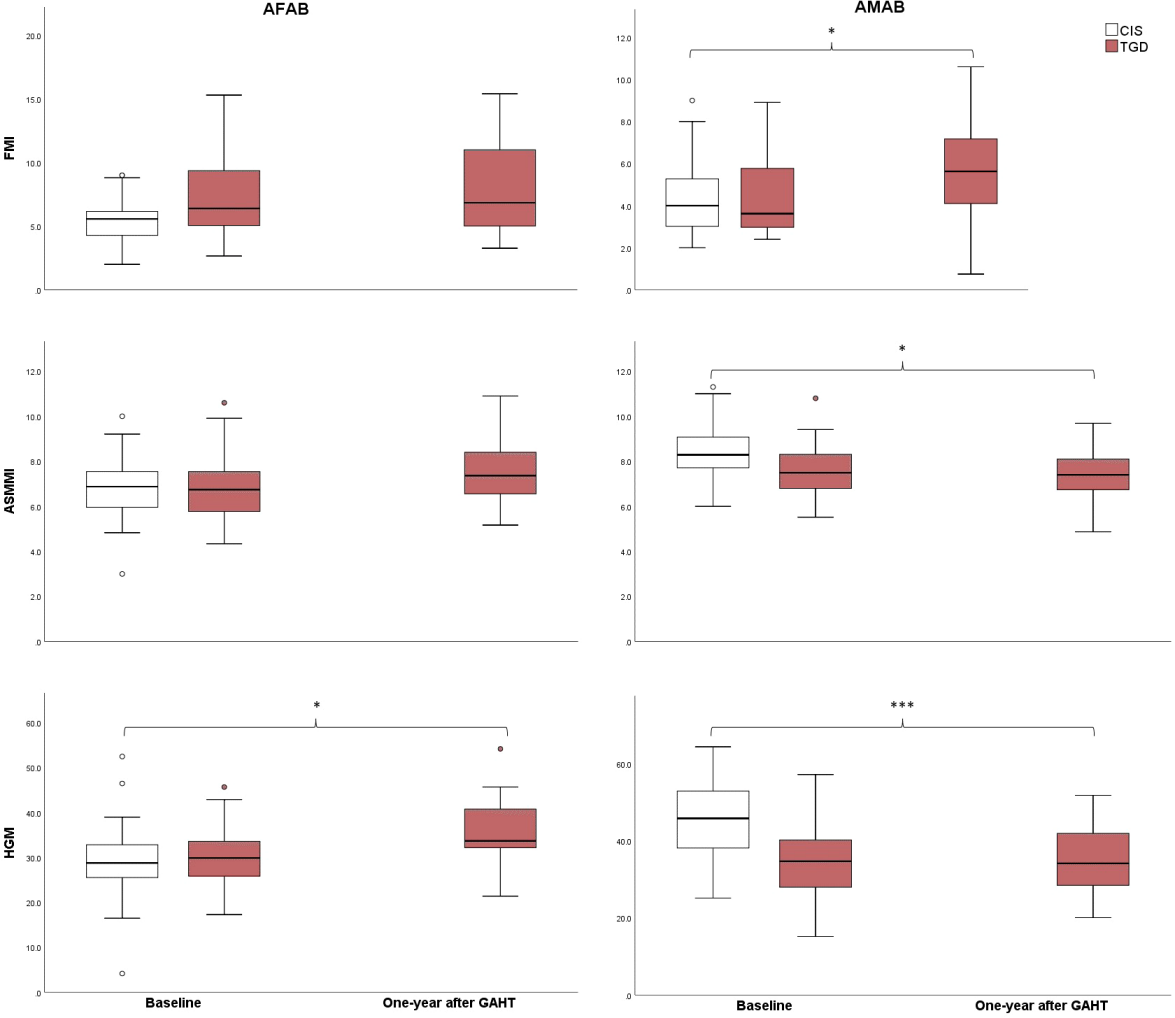
Changes in FMI, ASMMI, and muscle strength from baseline to one year after the initiation of GAHT. CIS, Cisgender; TGD, Transgender; AFAB, Assigned Female At Birth; AMAB, Assigned Male At Birth; FMI, Fat Mass Index; ASMMI, Appendicular Skeletal Muscle Mass Index; HGM, Handgrip max strength test. *p<0.05; ***p<0.001.

The key differences in body composition parameters during the first year of GAHT are outlined in **Table 4** and **Figure 1**. TGD AFAB individuals experienced an increase in muscle strength after one year of GAHT [32.24 (5.35) pre-GAHT vs. 34.91 (6.35) post-GAHT], with a 9% rise in lean mass and an 8% improvement in muscle strength (**Table 4**). However, their values for lean mass remained significantly lower compared to the cisgender AMAB group. Notably, TGD AFAB individuals exhibited a higher adipose component compared to cisgender peers. The pQCT analysis revealed a reduction in tibial fat area values, although these values remained higher compared to the cisgender counterparts. On the other hand, TGD AMAB individuals exhibited an increase in fat mass indices, particularly in fat percentage (+24%), FMI, and fat area measured by pQCT, along with a concurrent decrease in muscle mass indices [ASMMI: 8.07 (1.07) pre-GAHT vs. 7.37 (1.12) post-GAHT, p<0.05; muscle area: 7116.63 (1000.39) pre-GAHT vs. 6562.36 (945.93) post-GAHT, p<0.05]. These values were similar to those of cisgender AFAB individuals, except for muscle strength, which was higher in TGD AMAB individuals [34.91 (9.56) in TGD AMAB vs. 29.72 (7.58) in cis AFAB individuals]. Compared to cisgender individuals of the same sex at birth, TGD AFAB individuals showed a significant increase in muscle strength (p=0.03), while TGD AMAB individuals exhibited a significant increase in FMI (p=0.03), with muscle strength values remaining significantly lower than those of cisgender AMAB individuals (p<0.001) (**Figure 1**).

**Table 4:** Body composition-related variables after one year of GAHT, compared to the reference cisgender population.

Variable	TGD AFAB		AMAB Cisgender	TGD AMAB		AFAB Cisgender
	Baseline	1-year GAHT		Baseline	1-year GAHT	
**BMI**	23.75 (4.66)	24.86 (5.52)*	23.34 (2.48)^§^	23.28 (4.08)	23.26 (4.42)	21.79 (2.45)§§
**%fat**	29.57 (8.44)	27.66 (7.23)	18.01 (5.50)^§§§^	19.56 (6.74)	24.30 (7.95)***	24.66 (5.54)
**FMI [Kg/m^2^]**	7.57 (3.51)	7.48 (3.47)	4.41 (1.61)^§§§^	4.78 (2.30)	6.02 (2.60)***	6.02 (3.07)
**ASMMI [Kg/m^2^]**	7.17 (0.75)	7.39 (1.32)	8.48 (1.07)^§§§^	8.07 (1.07)	7.37 (1.12)*	6.88 (1.05)
**HGM [Kg_f_]**	32.24 (5.35)	34.91 (6.35)***	45.55 (34.91)^§§§^	32.21 (12.53)	34.91 (9.56)	29.72 (7.58)^§^
**Muscle area [cm^2^]**	6120.54 (1025.36)	5798.66 (1304.41)	7124.55 (1120.47)^§§§^	7116.63 (1000.39)	6562.36 (945.93)*	5608.70 (1877.44)
**Muscle density [mg/cm^3^]**	76.93 (2.13)	75.93 (4.10)	74.18 (2.21)^§§§^	76.38 (2.29)	73.56 (4.71)	74.79 (6.96)
**Fat area [cm^2^]**	3203.23 (1129.65)	2745.89 (1018.18)*	2028.82 (732.95)^§§§^	1713.96 (732.36)	2488.66 (582.79)**	2574.23 (954.82)

TGD, Transgender; AFAB, Assigned Female At Birth; AMAB, Assigned Male At Birth; BMI, Body Mass Index; FMI, Fat Mass Index; ASMMI, Appendicular Skeletal Muscle Mass Index; HGM, Handgrip max strength test. Asterisks refer to comparisons between transgender individuals before and after 1-year of GAHT: *p<0.05; **p<0.01; ***p<0.001. ^§^Refer to comparisons between transgender individuals after 1-year of GAHT and cisgender population: ^§^p<0.05; ^§§^p<0.01; ^§§§^p<0.001.Significant p-values are reported in bold.

**Figure 2:**
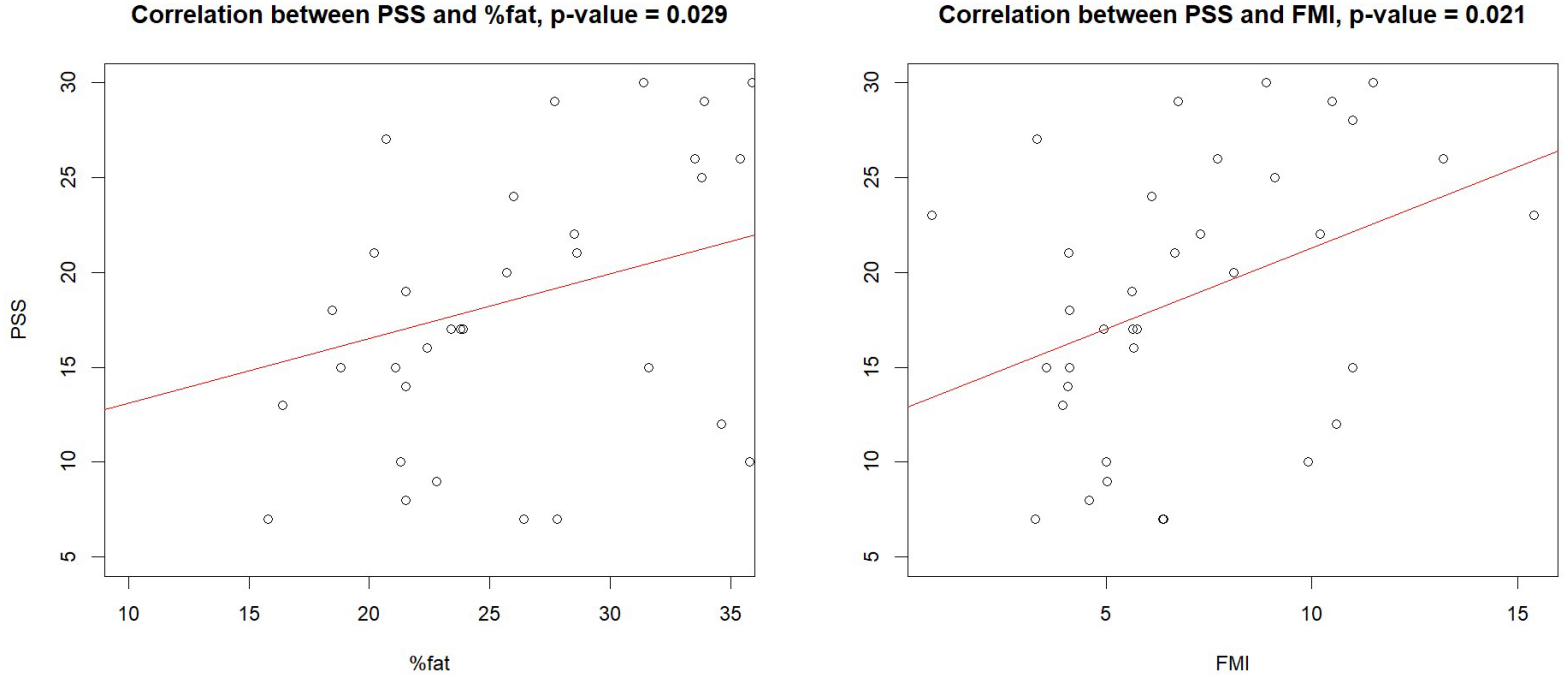
Correlations between PSS, fat, and FMI in the transgender population. FMI, Fat Mass Index; PSS, Perceived Stress Scale.

Examining the correlations between observed body changes and PHQ and PSS scores revealed that FMI and total fat percentage were positively correlated with PSS scores in the TGD population after one year of GAHT (**Figure 2**), especially among AFAB individuals (please see **Supplementary Table S1**). No similar correlations were found at baseline (please see **Supplementary Table S1**) or in the cisgender group. Additionally, PHQ scores were positively correlated with fat area (r=0.26, p=0.008), fat percentage (r=0.27, p=0.007) and FMI (r=0.23, p=0.02) only at baseline, while no correlations were observed with bone or body composition parameters during the first year of GAHT (please refer to **Supplementary Table S2**).

## Discussion

This study has demonstrated that GAHT significantly impacts bone density and body composition in TGD people after one year of treatment. Specifically, after one year of hormonal treatment, bone parameters at lumbar site improved and fat mass increased in TGD AMAB individuals, while muscle values increased in TGD AFAB individuals. However, during this period, PSS and PHQ scores did not change from baseline, remaining significantly elevated in the TGD sample. Additionally, in TGD individuals, PHQ and PSS scores were found to correlate with fat mass before and one year after the start of GAHT, respectively.

The evaluation of bone health in TGD individuals has been extensively studied. Sex hormones are critical in regulating bone turnover, inhibiting remodeling, resorption, and formation, with estrogen playing a central role in this process (21). Before the initiation of GAHT, research has consistently shown that TGD AMAB individuals tend to have lower bone parameters, such as BMD and z-scores, compared to their cisgender peers (5, 22). Consequently, under the influence of estrogen - which is crucial for bone growth in both length and width (23) - an improvement in bone density and structural parameters would be anticipated. Our findings confirm this trend, demonstrating significantly better lumbar BMD compared to baseline. However, BMD values in all examined regions remain significantly lower than those in cisgender women. These results align with previous studies (22, 24–26), further confirming the observed improvements in lumbar bone parameters, while changes at the femoral level remain less pronounced (27). Several theories have been proposed to explain the observed increase in bone values, with the osteoprotective effects of estrogen being a key factor. However, vitamin D likely also plays a role. Although our study did not find a statistically significant result, it is well-documented that TGD individuals, both AMAB and AFAB, typically have lower vitamin D levels before starting GAHT (7). One year after the initiation of GAHT, we observed a significant increase in vitamin D levels, aligning them with the levels of cisgender counterparts. This improvement is likely due to vitamin D supplementation, which is commonly prescribed during our endocrinological follow-ups when baseline levels are insufficient. Wiepjes et al. reported that BMD increased more in TGD AMAB individuals who used vitamin D supplements compared to those who did not, suggesting a similar impact in our study population (28). For TGD AFAB individuals, significant changes were observed after one year of GAHT, with a reduction in z-scores across all analyzed regions compared to baseline. However, no significant changes were noted in BMD or geometric parameters, except for an increase in CSAc compared to baseline. This finding is consistent with previous studies, which reported minimal changes in BMD in this population (22, 28, 29).

Sexual dimorphism in human body composition becomes particularly pronounced during puberty, driven by the action of sex hormones. Adult cisgender males typically have greater total lean mass and lower fat mass than cisgender females (30). These differences are further reflected in tissue distribution: cisgender males have more arm muscle mass and less limb fat, while central abdominal fat levels are comparable between the sexes (30). In contrast, cisgender females display a more peripheral fat distribution in early adulthood, which tends to shift with aging (30). Consequently, GAHT is expected to induce body composition changes toward their desired gender. In line with previous findings (6), our study demonstrates that in TGD AFAB individuals, GAHT leads to an increase in FFM and muscle strength, although muscle strength remains significantly lower compared to cisgender AMAB individuals, as measured by dynamometry. In terms of fat mass indices, we observed a reduction only at the tibial level compared to baseline, while fat percentage and FMI remain significantly higher than in cisgender men. Conversely, TGD AMAB individuals experience a notable increase in fat mass and a parallel decrease in lean mass after one year of GAHT, resulting in body composition values closely resembling those of cisgender AFAB individuals. These findings are in line with existing literature (5, 6, 9, 10, 31). However, muscle strength in TGD AMAB individuals remains significantly higher compared to cisgender AFAB counterparts. Our study stands out for its focus on the levels of stress and depression perceived by TGD individuals. Before the initiation of hormone therapy, stress and depression scores in the TGD cohort were significantly higher compared to the cisgender cohort and PHQ values were positively correlated with fat mass parameters. Stress levels did not show significant changes after one year of GAHT. Although evidence on its concrete impact on gender dysphoria and psychological functioning remains limited, it has been shown that gender affirming care can improve quality of life (1, 32). Previous studies have shown that psychiatric symptoms tend to decrease with appropriate GAMST, as well as with interventions aimed at reducing discrimination and gender minority stress (33–36). Fisher et al. reported that transgender individuals on GAHT show significantly lower levels of subjective gender dysphoria, body discomfort, and depressive symptoms compared to those not undergoing therapy (37). In the study by Grannis et al. on transgender boys, anxiety and depression severity was significantly lower in the testosterone-treated group compared to the untreated group, with a tendency towards lower suicidality and less body-related distress (36). Additionally, differences in depression and suicidality were directly related to body image dissatisfaction (36). The results of our study might be influenced by the timing of the assessment. According to Fisher et al., global levels of gender dysphoria tend to increase at 12 months of GAHT (37). This may be due to the fact that most body changes in both AFAB and AMAB TGD individuals typically occur between 2 and 5 years after starting hormone therapy (2). Additionally, other factors not directly related to the immediate effects of GAHT could also play a role. In fact, while body changes from GAHT on perceived stress measure are linked to psychological improvements, issues related to social and legal recognition often increase over time (32, 37, 38). Socio-cultural factors, such as misgendering—where individuals are not recognized or addressed by their unwanted name, pronoun, or gender—may continue to contribute to gender minority stress (16, 17). In Italy, for instance, individuals must wait one year on GAHT before they can apply for legal gender recognition and, if desired, gender-affirming surgeries (39). This waiting period can create a mismatch between gender expression and official documents, leading to forced coming out situations and increasing dysphoria related to the socio-legal aspects of gender incongruence. Consequently, while hormone therapy may alleviate gender dysphoria, it may not necessarily reduce gender minority stress. Studies suggest that TGD individuals who have updated their gender markers on official documents tend to experience better mental health (1, 35).

The persistent correlation after one year of GAHT between self-reported stress levels and fat mass parameters—though involving PSS rather than PHQ—highlights the potential impact of psychological well-being on the health of TGD individuals. This is particularly relevant in the TGD AFAB subgroup. We believe this may be linked to the fact that a gynoid fat distribution, with increased fat accumulation in the hips and potentially the breasts, could contribute to higher gender dysphoria and, consequently, increased stress. Additionally, a higher fat mass might obscure the masculinizing effects of therapy, such as muscle growth and changes in facial features, potentially perpetuating a cycle of dissatisfaction. Moreover, the presence of substantial adipose tissue may diminish the effectiveness of testosterone at the tissue level. Adipose tissue contains aromatase enzymes, which can convert exogenous testosterone into estrogen (40), potentially reducing the efficacy of GAHT. Previous studies have also reported a significant association between weight stigma, gender identity, and mental health in youth (41, 42), suggesting a possible convergence of gender minority stress with weight stigma. Furthermore, the higher incidence of eating disorders among TGD individuals compared to the cisgender population could contribute to increased fat mass and associated stress levels (42–44).

Some limitations of the study should be acknowledged. The first limitation is the small sample size of TGD individuals who completed the one-year follow-up after GAHT, which has constrained the statistical power of our analyses. Additionally, the exclusive use of self-assessment questionnaires to evaluate stress, without incorporating more comprehensive gender minority stress screening methods, may have reduced the significance of the collected data. Moreover, current guidelines recommend performing DXA follow-up scans after 18-24 months; however, we conducted our assessment 12 months after the start of GAHT. This decision was primarily driven by the desire to closely monitor the early effects of hormone therapy on musculoskeletal health and to consider the potential impact of perceived stress on these systems. In fact, scientific literature reports significant differences in bone density and body composition after 12 months of GAHT (29). On the other side, a key strength of our study is the thorough analysis of body composition parameters. We employed pQCT, allowing for more detailed insights into bone and body composition. Moreover, this study is pioneering in exploring the correlation between stress and body composition, making a significant contribution to our understanding of how psychological factors interact with physical health in TGD individuals.

In conclusion, our study highlights the potential impact of perceived stress on health, with particular emphasis on its possible influence on body changes associated with GAHT. We hope that our study, the first to attempt to correlate bone and body composition parameters with perceived stress levels, can serve as a foundation for further research exploring the impact of stress on the health of TGD people. Research needs further investigation that takes into account the use of multi-method perspectives that investigate multiple dimensions to understand the role that risk factors, as well as protective factors, have on individual’s overall well-being.

## Data Availability

The raw data supporting the conclusions of this article will be made available by the authors, without undue reservation.
