# Supplementary material for: Body composition and perceived stress levels in transgender individuals after one year of gender affirming hormone therapy
Source: Front Endocrinol (Lausanne). 2024 Nov 28;15:1496160. doi: 10.3389/fendo.2024.1496160 (PMC11634618; doi:10.3389/fendo.2024.1496160)
Supplement: Supplementary file 1 [file Table1.docx]

**Supplementary Table 1. Simple correlations between PSS at baseline and after one-year of GAHT, and bone and body composition parameters in transgender people.**

|  |  | **BMD total hip** | **BMD femur neck** | **BMD lumbar spine** | **CSA** | **BMDtrb** | **BMCcrt** | **CSAc** | **Muscle density** | **Muscle area** | **Fat area** | **%fat** | **FMI** | **ASMMI** |
| --- | --- | --- | --- | --- | --- | --- | --- | --- | --- | --- | --- | --- | --- | --- |
| ***All sample*** | ***At baseline*** | -0.12, p=0.21 | -0.06, p=0.52 | 0.001, p=0.99 | -0.06, p=0.52 | -0.02, p=0.81 | -0.14, p=0.16 | 0.035, p=0.72 | -0.18, p=0.08 | 0.02, p=0.80 | 0.17, p=0.08 | 0.13, p=0.18 | 0.14, p=0.13 | 0.03, p=0.72 |
|  | ***One-year after GAHT*** | 0.15, p=0.36 | 0.20, p=0.21 | 0.15, p=0.35 | -0.06, p=0.74 | -0.03, p=0.84 | 0.14, p=0.25 | 0.10, p=0.56 | -0.18, p=0.32 | -0.19, p=0.30 | 0.37, p=0.05 | **0.36, p=0.029** | **0.38, p=0.021** | -0.06, p=0.73 |
| ***AFAB*** | ***At baseline*** | -0.13, p=0.52 | 0.07, p=0.72 | 0.28, p=0.13 | -0.03, p=0.87 | -0.01, p=0.96 | -0.10, p=0.62 | 0.15, p=0.48 | -0.24, p=0.23 | 0.20, p=0.32 | 0.11, p=0.59 | 0.21, p=0.26 | 0.23, p=0.22 | 0.06, p=0.76 |
|  | ***One-year after GAHT*** | 0.17, p=0.42 | 0.25, p=0.23 | 0.21, p=0.32 | 0.14, p=0.59 | 0.10, p=0.69 | -0.11, p=0.67 | 0.18, p=0.48 | -0.02, p=0.93 | -0.38, p=0.16 | 0.34, p=0.21 | **0.68, p<0.001** | **0.58, p=0.006** | -0.18, p=0.43 |
| ***AMAB*** | ***At baseline*** | -0.44, p=0.04 | -0.18, p=0.42 | -0.37, p=0.09 | -0.08, p=0.73 | -0.32, p=0.14 | -0.34, p=0.12 | -0.11, p=0.61 | 0.29, p=0.19 | 0.16, p=0.50 | 0.29, p=0.19 | -0.17, p=0.44 | -0.23, p=0.30 | 0.01, p=0.95 |
|  | ***One-year after GAHT*** | 0.10, p=0.72 | 0.06, p=0.82 | 0.03, p=0.90 | 0.02, p=0.94 | -0.02, p=0.93 | -0.21, p=0.42 | -0.16, p=0.54 | 0.21, p=0.42 | -0.025, p=0.34 | -0.17, p=0.52 | -0.13, p=0.64 | -0.12, p=0.67 | 0.22, p=0.42 |

*Abbreviation*: PSS = Perceived Stress Scale; BMD=Bone Mineral Density; CSA=Cross-Sectional Area; trb= trabecular; CSAc= Cross-Sectional Area Cortical; FMI = Fat Mass Index; ASMMI = Appendicular Skeletal Muscular Mass Index.

**Supplementary Table 2. Simple correlations between PHQ at baseline and after one-year of GAHT and bone and body composition parameters in transgender people.**

|  |  | **BMD total hip** | **BMD femur neck** | **BMD lumbar spine** | **CSA** | **BMDtrb** | **BMCcrt** | **CSAc** | **Muscle density** | **Muscle area** | **Fat area** | **%fat** | **FMI** | **ASMMI** |
| --- | --- | --- | --- | --- | --- | --- | --- | --- | --- | --- | --- | --- | --- | --- |
| ***All sample*** | ***At baseline*** | -0.02, p=0.81 | -0.005, p=0.96 | -0.02, p=0.85 | -0.02, p=0.87 | -0.007, p=0.95 | -0.03, p=0.75 | -0.02, p=0.83 | -0.07, p=0.48 | 0.02, p=0.85 | **0.26, p=0.008** | **0.27, p=0.007** | **0.23, p=0.02** | -0.03, p=0.74 |
|  | ***One-year after GAHT*** | 0.13, p=0.14 | 0.25, p=0.11 | 0.11, p=0.47 | -0.08, p=0.65 | -0.15, p=0.41 | -0.14, p=0.44 | 0.18, p=0.31 | -0.01, p=0.96 | -0.33, p=0.08 | 0.22, p=0.24 | 0.17, p=0.32 | 0.23, p=0.17 | -0.06, p=0.74 |
| ***AFAB*** | ***At baseline*** | -0.03, p=0.88 | 0.15, p=0.45 | 0.20, p=0.29 | 0.21, p=0.30 | -0.07, p=0.73 | 0.06, p=0.77 | 0.09, p=0.64 | -0.08, p=0.68 | 0.27, p=0.29 | 0.22, p=0.29 | 0.35, p=0.06 | 0.30**,** p=0.11 | 0.12, p p=0.52 |
|  | ***One-year after GAHT*** | 0.09, p=0.65 | 0.24, p=0.22 | 0.12, p=0.55 | 0.13, p=0.60 | -0.003, p=0.99 | -0.09, p=0.72 | 0.17, p=0.52 | -0.05, p=0.08 | -0.28, p=0.27 | 0.20, p=0.48 | 0.29, p=0.19 | 0.35, p=0.11 | -0.04, p=0.85 |
| ***AMAB*** | ***At baseline*** | -0.27, p=0.28 | -0.26, p=0.28 | -0.39, p=0.08 | -0.14, p=0.53 | 0.08, p=0.71 | -0.13, p=0.55 | -0.03, p=0.87 | 0.44, p=0.04 | -0.21, p=0.36 | 0.14, p=0.55 | 0.02, p=0.92 | -0.19, p=0.38 | -0.39, p=0.07 |
|  | ***One-year after GAHT*** | 0.18, p=0.49 | 0.27, p=0.32 | 0.08, p=0.75 | -0.32, p=0.23 | -0.25, p=0.37 | 0.09, p=0.76 | 0.32, p=0.25 | **-0.61, p=0.02** | 0.29, p=0.31 | 0.27, p=0.37 | -0.08, p=0.64 | -0.12, p=0.67 | 0.22, p=0.42 |

*Abbreviation*: PHQ = Patient Health Questionnaire; BMD=Bone Mineral Density; CSA=Cross-Sectional Area; trb= trabecular; CSAc= Cross-Sectional Area Cortical; FMI = Fat Mass Index; ASMMI = Appendicular Skeletal Muscular Mass Index.
